# The Effect of Carbonic Maceration during Winemaking on the Color, Aroma and Sensory Properties of ‘*Muscat Hamburg*’ Wine

**DOI:** 10.3390/molecules24173120

**Published:** 2019-08-28

**Authors:** Yu-Shu Zhang, Gang Du, Yu-Ting Gao, Li-Wen Wang, Dan Meng, Bing-Juan Li, Charles Brennan, Mei-Yan Wang, Hui Zhao, Su-Ying Wang, Wen-Qiang Guan

**Affiliations:** 1 Tianjin Key Laboratory of Food Biotechnology, College of Biotechnology and Food Science, Tianjin University of Commerce, Tianjin 300134, China; 2 Centre for Food Research and Innovation, Department of Wine, Food and Molecular Bioscience, Lincoln University, Lincoln 7647, New Zealand

**Keywords:** Muscat Hamburg wine, carbonic maceration, color, aroma, sensory evaluation

## Abstract

This is the first study on the effect of carbonic maceration on the quality (color, aroma profile and sensory properties) of Muscat Hamburg, contrasting two winemaking procedures used in Tianjin (classical white and red-winemaking techniques). The values of C* (psychometric chroma), a* (measure of redness) and b* (measure of yellowness) were significantly higher (*p* < 0.01) in the carbonic macerated wine than in red wine. However, there were no visual differences in color, and classical red wine and carbonic macerated wine had similar *h* (hue angle) values and located in the red region. Thirty-two aromatic compounds were identified and quantified in Muscat Hamburg wines. The content of volatile compounds (6384.97 μg/L) was significantly higher (*p* < 0.001) in the carbonic macerated Muscat Hamburg wine than in the other kinds of wine. This result led to the carbonic macerated wine having the highest odor activity values (OAVs) and sensory evaluation scores (86.8 points), which correlates with an “excellent” sensory perception. This study demonstrated that carbonic maceration significantly improved the quality of Muscat Hamburg wine based on volatile analysis and sensory evaluation compared with other conventional methods. Therefore, carbonic maceration could be well suited for making Muscat Hamburg wine.

## 1. Introduction

Muscat Hamburg grapes (*Vitis vinifera* L. cv. Muscat Hamburg) have been grown in Tianjin for more than one hundred years due to the advantageous properties of this type of grape including: wide adaptability, strong disease resistance, high yield, easy management and strong flavor. Muscat Hamburg grapes are commonly consumed as both table grapes and grapes for winemaking, and the grape flavor characteristics make them very popular in the region. Muscat Hamburg wine (a dry white wine) was initially produced by the Tianjin Dynasty Winery in the 1980s. This type of wine was highly awarded in worldwide wine competitions. Since then, the production of Muscat Hamburg wine has dramatically increased, and currently, approximately 5000 tons is produced every year (95% of which is white wine, 2% is red wine and 3% is sparkling wine).

There are two main and popular techniques used in making fresh Muscat Hamburg wines in China including classical white-winemaking and red-winemaking techniques. However, these two types of Muscat Hamburg wine cannot meet the demand of consumers for the high-quality wine. The reason for the lack of consumer appreciation of these wines as high-quality wines appears to be due to the fact that the content of phenolic substances in white wine is lower, and the wine has a poorer mouthfeel as well as a lower beneficial compounds (polyphenols) due to the reduced involvement of the grape skin in the white-winemaking process [1,2]. On the other hand, the red-winemaking process, which includes daily (1–3 times) pumping over in air, results in a lower content of aroma compounds. Indeed, the effect of the phenolic compounds in wine quality has been studied extensively in recent years in many different types of wine regions [3,4]. Therefore, the demand from the market has driven research into elucidating the factors that affect the quality of wines and developing new winemaking techniques and new types of wine. Wine quality is mainly assessed by three factors, the color, aroma and taste of the wine. Different fermentation technologies can be used to improve these quality characteristics to suit consumer preferences.

Carbonic maceration is widely used to produce wine with novel characteristics. The technique has been used in France for more than 80 years [5], and it exploits a process that takes place naturally inside grape berries stored intact in an anaerobic environment. After carbonic maceration, the final product has a richer flavor and superior aromatic qualities [6] with a higher content of phenolic compounds [5], which are desirable to consumers. Carbonic maceration techniques have been used with many grape varieties, including Gamay, Tempranillo, Monastrell, Airén, Syrah, and Tinta Miúda grapes [5,6,7,8,9,10]. However, to the best of our knowledge, there are no studies applying this technique to Muscat Hamburg grapes.

Therefore, the main objective of this study is to determine the influence of carbonic maceration compared with classical red and white wine fermentation techniques on the color, aroma and sensory properties of Muscat Hamburg wine compared with classical red and white wine fermentation techniques. Results from this work will improve our comprehension in carbonic maceration wine fermentation technique used in Muscat Hamburg grapes to enhance the quality of Muscat Hamburg wine.

## 2. Results and Discussion

### 2.1. General Composition of Wine

After the fermentations were completed (final concentration of sugar < 2 g/L), the general compositions of the wines were measured, and the results are summarized in Table 1.

In general, Muscat Hamburg wines made by different winemaking techniques did not have significantly different (*p* < 0.05) alcohol concentrations, contents of reducing sugar, free SO_2_ levels, total acidities and dry extract contents. The Muscat Hamburg wines made by carbonic maceration exhibited higher pH values than the wines obtained by classical red- and white-winemaking processes. This observation can be attributed to two factors. First, the malic acid content was reduced in the carbonic macerated wine because of the carbonic maceration technique itself; depending on the grape variety and fermentation temperature, greater than 15%–60% of the malic acid is metabolized during the carbonic maceration process [11,12]. The second reason could be that the lower fermentation temperature (13–15 °C) after carbonic maceration led to precipitation of calcium tartrate and potassium tartrate. These factors resulted in the Muscat Hamburg wines produced by carbonic maceration being more acidic than the wines produced by the other two techniques.

### 2.2. Color Measurements

The values of psychometric lightness (L*), hue angle (*h*), and psychometric chroma (C*) were analyzed, and the values in three-dimensional color space, a* (measure of redness) and b* (measure of yellowness), of the two types of red wines (R and CM) were also determined, as highlighted in Table 2.

The L* values were very high in the Muscat Hamburg wines produced by the red-winemaking and carbonic maceration techniques (96.41 and 96.79, respectively). The values of C* (41.26), a* (43.25) and b* (0.84) were significantly higher (*p* < 0.01) in the wine produced by carbonic maceration than in the wine produced by red-winemaking techniques (35.67, 37.65 and 0.75, respectively). However, visually, the carbonic macerated wine did not have a more vivid color. This fact can be attributed to its greater lightness, which affects the saturation (S*_uv_ = C*_uv_/L*) more than the chroma, making this wine less saturated and preventing us from seeing a purer color [9]. The classical red wine and carbonic maceration-derived samples had similar *h* (hue angle) values (located in the red region), and the colors of these two kinds of wine were not significantly different. Our results agree with the previous finding that the wines made by carbonic maceration had higher chroma values than wines obtained by classical red-wine vinification techniques [13]. Moreover, this result can be explained by the carbonic maceration leading to a lower anthocyanin content, mainly monoglucosides and total phenols [13], which is related to lighter wines and less saturated but more colorful samples (higher chroma values).

### 2.3. Aromatic Profile Analysis

The results of the volatile compounds, odor descriptors and thresholds are shown in Table 3. In this study, the olfactory perception thresholds and odor descriptors of compounds in wine were taken from the available literature [14,15,16,17,18]. Thirty-two compounds, including alcohols (two), aldehydes and ketones (five), terpenes (10), acids (two) and esters (13), were identified and quantified in Muscat Hamburg wine. According to the quantitative data, the concentration of total volatiles in the wines ranged from 3614.66 to 6384.97 μg/L. The different winemaking methods significantly (*p* < 0.001) influenced the concentration of volatiles in the wines. Among the three kinds of wines, the Muscat Hamburg wine prepared by carbonic maceration contained the highest concentration of volatiles (6384.97 μg/L), followed by the wines prepared by classical white-winemaking (4251.65 μg/L) and red-winemaking (3614.66 μg/L) techniques. This result may be due to the occurrence of anaerobic fermentation in the absence of oxygen and saturation with CO_2_, and the profile of volatile organic compounds (VOCs) changes significantly. Many studies have reported that carbonic maceration resulted in higher concentrations of VOCs, especially esters and fatty acids [19].

Esters were the most abundant type of aromatic compounds in the wines (77.1%–82.15% (*w*/*w*)), followed by terpenes and aldehydes. The concentrations of total esters in the wines ranged from 2969.24 to 5221.26 μg/L. Esters are largely responsible for the fresh and fruity aromas of wines and are mainly produced during alcoholic fermentation [20]. The different winemaking technologies significantly (*p* < 0.001) influenced the concentration of esters in the wines. The concentration of esters in the Muscat Hamburg wine made by carbonic maceration was higher than that in the Muscat Hamburg wine made by classical white and red-winemaking techniques. This result was attributed to a much higher content of ethyl esters (ethyl octanoate, ethyl hexanoate and ethyl decanoate) and acetate esters (hexenyl acetate, isoamyl acetate and phenylethyl acetate). Ethyl esters, which are derived from medium-chain fatty acids, are responsible for the fruity character of wines, the most representative aroma family in all wines. Ethyl octanoate, ethyl hexanoate and ethyl decanoate, which are associated with pleasant notes “pear”, “banana” and “fruity”, were the most abundant ethyl esters. Acetate esters, which are formed from acetic acid and higher alcohols, have a greater influence on the perceived aroma than ethyl esters [21]. Among the quantified acetate esters, three compounds (hexenyl acetate, isoamyl acetate and phenylethyl acetate) were associated with the positive attributes “fruits”, “banana” and “floral” in the Muscat Hamburg wines. The contents of ethyl esters and acetate esters were significantly higher (*p* < 0.001) in the Muscat Hamburg wine made by carbonic maceration. In addition, our results, which indicate higher levels of ethyl octanoate, ethyl hexanoate and ethyl decanoate in the Muscat Hamburg wines, are consistent with the results of previous studies on Muscat wines [22].

The concentrations of terpenes in the wines ranged from 405.1 to 787.9 μg/L. (±)-β-Citronellol, linalool and limonene were the greatest contributors to the higher concentration of terpenes in Muscat Hamburg wine. Similar results were observed in a previous study that reported that aromatic varieties, such as Muscat Hamburg, Gewürztraminer, Irsai Oliver and Rhine Riesling, had the highest terpenoid concentrations [23]. In addition, the contents of terpenes were significantly (*p* < 0.001) higher in the wine produced by carbonic maceration (787.9 μg/L) compared with the levels in the wines produced by white- and red-winemaking techniques (678.96 μg/L and 405.1 μg/L, respectively). This result agrees with the previous finding that both the free and bound terpenoid contents were markedly higher when using carbonic maceration at higher temperatures (32 °C) [24]. These terpenes played a key role in the OAVs of Muscat Hamburg wines due to the low thresholds of these terpene compounds, such as (±)-β-citronellol, linalool and limonene. Numerous studies have reported that terpene compounds can be used analytically for varietal characterization. Terpene compounds are secondary plant metabolites derived from acetyl Co-A. Terpenes are also synthesized by microorganisms; however, the synthesis of terpenes by *Saccharomyces cerevisiae* has not been observed. Terpenes are important components of varietal aroma and are not influenced by yeast metabolism during fermentation [25].

Low numbers of aldehydes (four), ketones (one), acids (two) and alcohols (two) were detected in the Muscat Hamburg wines, and their concentrations were much lower than those of the other aroma compounds. Due to its low odor threshold (0.05 µg/L), β-damascenone contributed substantially to the OAVs. Alcohols are formed by the degradation of amino acids, carbohydrates and lipids [26]. The production of fatty acids has been reported to be dependent on the composition of the must and the fermentation conditions [27]. Fatty acids are associated with fatty olfactory notes [20].

Among the 32 volatile compounds identified and quantified in the wines, only 17 compounds were detected at levels above their odor thresholds (OAVs > 1), and these compounds included an alcohol, an aldehyde, a ketone, terpenes (five) and esters (nine). Ethyl octanoate had the highest OAV (> 300), followed by β-damascenone (OAV > 100) and then (−)-rose oxide. (±)-β-Citronellol, linalool, limonene, isoamyl acetate and ethyl hexanoate were present at concentrations above the perception threshold (OAVs > 10). 3-Methyl-1-butanol, nonanal, geraniol, (*Z*)-3-hexenyl acetate, ethyl butyrate, ethyl caproate, hexenyl acetate, ethyl decanoate and phenylethyl acetate were also present at concentrations above the perception threshold (OAVs > 1).

An aromatic series can be defined as a group of volatile compounds with similar odor descriptors. Based on its odor descriptors, each compound was assigned to one or more aromatic series: solvent, floral, sweet, green (vegetal or herbaceous), fatty or fruity, which were chosen because these terms are commonly used to describe young wines [28]. The value of each aromatic series was obtained as the sum of the OAVs of the compounds in the series. Therefore, the contribution of a specific compound to each series can be determined. This procedure allows the quantitative information obtained by chemical analysis to be related to sensory perception.

**Figure 1:**
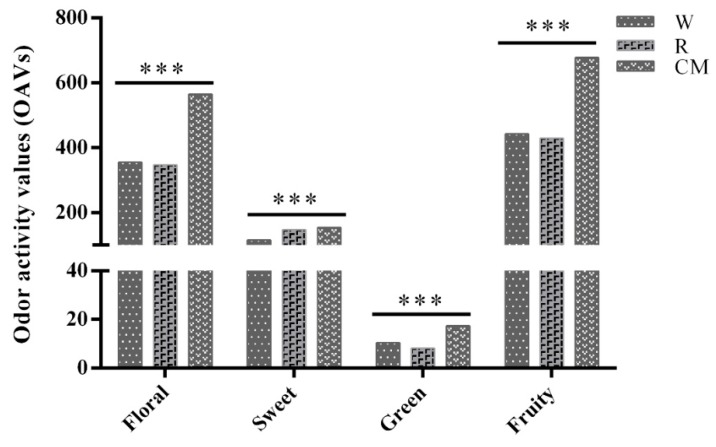
The impact of different fermentation technologies on the odor activity values (OAVs) of the odorant series for Muscat Hamburg wine. *** *p* < 0.001.

The impact of different fermentation technologies on the OAVs of the odorant series for Muscat Hamburg wine is summarized in Figure 1. The fruity and floral series had the greatest impacts on the OAVs of the wine, followed by the sweet and green series. The solvent and fatty series were not detected in the wines. The contributions of all the odorant series were significantly different in the different wines (*p* < 0.001). The OAVs of the Muscat Hamburg wine made by carbonic maceration were significantly higher than those of the wines made by classical white- and red-winemaking processes (*p* < 0.001). Carbonic maceration exposes intact grape berries to an oxygen-deprived environment, allowing enzymatic fermentation to occur within the berries, typically resulting in richly aromatic wines with a fruity bouquet and palate softness [11]. These results provide clear evidence that carbonic maceration can be adopted by wineries to obtain Muscat Hamburg wines with superior quality.

### 2.4. Sensory Evaluation

The average scores of all the types of Muscat Hamburg wine in the sensory evaluation are shown in Table 4. Wines with higher scores have more desirable attributes. Significant differences (*p* < 0.001) between the different wines were found in the aroma analysis (aroma purity, intensity and quality), taste analysis (taste purity, taste duration and taste quality) and harmony. Carbonic macerated Muscat Hamburg wine received the highest scores in aroma analysis, taste analysis and harmony (26.3, 37.5 and 10.2, respectively), followed by white Muscat Hamburg wine (24.2, 35.4 and 9.5, respectively) and red Muscat Hamburg wine (22.4, 34.1 and 8.6, respectively). The results of aroma analysis were in accordance with the previous quantitative analysis of aroma compound, which showed that Muscat Hamburg wine prepared by carbonic maceration had the highest content of esters and terpenes. The lower content of phenols, which greatly influence the taste and harmony of red wine, in the red Muscat Hamburg wine could by why this sample received the lowest scores in the taste and harmony categories of the sensory evaluation [29]. Carbonic macerated Muscat Hamburg wine received the highest score overall with 86.8 points and was rated as “excellent”. The classically prepared white wine, which received 82.1 points, was listed second and rated as “very good”, while the classically prepared red wine received 77.6 points and was rated as “good”. These results indicate carbonic maceration produces wines with distinctive of properties, superior quality, and a harmonious balance compared with wines produced by conventional techniques [11,30].

In general, carbonic macerated Muscat Hamburg wine received highest score, it is related to unique aroma, such as floral, fatty and fruity. The floral aroma is mainly determined by caryophyllene and α-terpineol, the fatty one may be connected with n–decanoic acid and (*E*)-2-nonenal, while ethyl laurate have a major role in the fruity aromatic series. Other aroma compounds in Muscat Hamburg wine have some effect of odor to a certain degree. Ultimately, the highest sensory evaluation is connected closely with the aroma compounds present.

## 3. Materials and Methods

### 3.1. Grape Sampling

The grapevines were grown in Chadian vineyard (38°83′–39°54′ N, 116°23′–117°45′ E), Hangu County, Tianjin, China. The vineyard had an annual accumulated temperature of 3900–4200 °C and an annual rainfall of 580–720 mm. The soil in the vineyard is mainly clay, and the row width and vine spacing were 2.5 and 1.0 m, respectively. The ten-year-old, own-rooted Muscat Hamburg vines were watered by a drip irrigation system. Grape samples were harvested at 22 °Bx and were of similar size and had no physical damage or infections.

### 3.2. Yeast Strains

*Saccharomyces cerevisiae* BH8 was used in this study. In a previous study, we found that the use of *S. cerevisiae* BH8 could increase the content of aroma compounds [31]. *S. cerevisiae* wine yeast strain BH8 was isolated from the ‘Beihong’ wine grape variety (‘*Muscat Hamburg*’ × ‘*V. amurensis*’) and cultivated by the Institute of Botany, the Chinese Academy of Sciences, Beijing, China [32]. This strain was identified as *S. cerevisiae* by color and colony topography on WL Nutrient Agar [33] as well as by DNA sequence analysis conducted at the Institute of Microbiology, Chinese Academy of Sciences, Beijing, China.

### 3.3. Fermentation Experiments

Three different winemaking methods were used in this study (classical white-winemaking, classical red-winemaking and carbonic maceration methods). Muscat Hamburg grapes were hand-harvested in 2014. For the classical white-winemaking method, the Muscat Hamburg grapes were immediately destemmed and gently crushed. Musts were clarified by cold-settling for 18 h at 10 °C to separate the clear juice from the sediment and the loading volume was about 90% of the tank capacity. The fermentation temperature was 13–15 °C, and the alcoholic fermentation lasted for 12 days. In classical red-winemaking method, the Muscat Hamburg grapes were destemmed and subjected to a short cold maceration (4 h at 10 °C) prior to the winemaking process and the loading volume was around 80% of the tank capacity. The fermentation temperature was controlled at 25–28 °C. In addition, during the fermentation process, half of the total volume of wine was pumped over in air 1–3 times per day depending on the kinetics of the fermentation. After the alcoholic fermentation was complete (7 days), only free-run must was used for bottling. For the carbonic maceration process, the intact grapes with stems were placed into a stainless-steel tank that had been saturated with food-grade carbon dioxide and hermetically closed. The tanks were saturated with carbon dioxide every 12 h. The temperature was controlled at 28 °C for 12 days. After carbonic maceration, only the must obtained by gentle pressing was used for fermentation. The fermentation temperature was controlled at 13–15 °C, and the alcoholic fermentation lasted for 7 days.

All grape must samples were treated with sulfur dioxide (sulfited) to a final concentration of 40 mg/L, and the final pH value of the must was adjusted to 3.3 by aseptically adding tartaric acid (85%, *w*/*v*). This organic acid was selected because it is normally found in grapes and wines and is rarely metabolized by ascomycetous yeasts. The initial yeast inoculums were 1 × 10^6^ cells/mL from cultures grown overnight in YPD medium (1% (*w*/*v*) yeast extract, 2% (*w*/*v*) peptone and 2% (*w*/*v*) glucose).

All the fermentations were carried out in 25-L stainless-steel tanks with at least three replicates. A stainless-steel mesh screen was fixed at the bottom of the 25-L stainless-steel tank during the carbonic maceration process to separate the intact grapes from the must. The fermentation temperature and must density were monitored periodically during the fermentation processes. After alcoholic fermentation, the wines were decanted into secondary tanks, cold stabilized for 3 weeks at 4 °C, and then bottled. The Muscat Hamburg wine samples were labeled as follows: W (classical white-winemaking process), R (classical red-winemaking process) and CM (carbonic maceration winemaking process).

### 3.4. General Enological Parameters

The general parameters of the wine were analyzed according to the Office International de la Vigne et du Vin [34]. The following parameters were analyzed: alcohol percentage, reducing sugars, pH value, total acidity, free SO_2_ and dry extract.

### 3.5. Color Measurements

After bottling and storage at 16–18 °C for one month, the colors of the wine were assessed. Color measurements were performed according to the CIELAB 76 convention [10] by determining the transmission data at wavelengths from 380 to 770 nm at 10-nm intervals. The cylindrical coordinates, L* (psychometric lightness), C* (psychometric chroma) and *h* (hue angle) values, were obtained using the Triest 1.0 program. The values in three-dimensional color space, a* (measure of redness) and b* (measure of yellowness), were calculated as described.

### 3.6. Volatile Compounds Analysis

The aroma compounds were extracted from the wine samples by headspace solid-phase microextraction (HS-SPME) and analyzed using gas chromatography-mass spectrometry (GC-MS) as described by Zhang et al. [35]. A 5-mL sample of wine was mixed with 1 g of NaCl in a 15-mL sample vial. The vial was tightly capped with a PTFE-silicon septum and heated at 40 °C for 30 min on a heating platform with agitation at 400 rpm. An SPME fiber (50/30 μm DVB/Carboxen/PDMS, Supelco, Bellefonte, PA, USA), preconditioned according to the manufacturer’s instructions, was then inserted into the headspace and left for 30 min with continuous heating and agitation using a magnetic stirrer to achieve suitable extraction.

The GC-MS system used in this study was an Agilent 6890 GC equipped with an Agilent 5975 mass spectrometer (Beijing, China). The column was a 60 m × 0.25 mm HP-INNOWAX capillary column with 0.25-μm film thickness (J & W Scientific, Folsom, CA, USA). Helium was used as the carrier gas at a flow rate of 1 mL/min. Samples were injected by placing the SPME fiber in the GC inlet for 25 min with the splitless GC inlet mode. The oven was initially held at 50 °C (for 1 min), heated to 220 °C at a rate of 3 °C/min and then held at 220 °C for 5 min. The samples were analyzed by mass spectrometry in electron impact mode (MS/EI) at 70 eV over the *m*/*z* range of 20 to 450 U. The mass spectrophotometer was operated in selective ion mode with automatic peak selection, and the area of each peak was determined by analysis with ChemStation software (Agilent Technologies). Each analysis was repeated three times.

All standard solutions were purchased from Sigma-Aldrich (Beijing, China) and Fluka (Buchs, Switzerland). All standards had purities above 97%. Sample solutions were prepared using the methods reported by Howard et al. [36]. 4-Methyl-2-pentanol was used as internal standard. Five-point calibration curves for the quantification of each compound were prepared using the method described by Ferreira et al. [37]. These curves were also used as a reference to determine the appropriate concentration range for preparing standard solutions. The regression coefficients of the calibration curves were above 97% (*w*/*w*).

### 3.7. OAV Calculation

The aromatic profiles of the wines were evaluated based on their “odor activity values” (OAVs). This aromatic index allows the degree of participation of each compound in the final aroma to be determined. In this sense, only compounds with OAVs > 1 were considered active odorants. OAVs were calculated using the equation OAV = c/t [38], where c is the concentration of the individual compound in the wine, and t is the olfactory perception threshold of the compound in the wine.

### 3.8. Sensory Evaluation

After bottling and storage at 16–18 °C for two months, wine samples were subjected to sensory evaluation. The panelists were selected as described by Tomasino et al. [39]. The wine samples were evaluated by a trained and experienced panel composed of 15 tasters (10 male, 5 female) from different wineries in Tianjin, China. The wine samples (30 mL) were randomly labeled and arbitrarily presented to the panel in a standard wine tasting room in Tianjin Dynasty Winery. The evaluation consisted of describing the appearance, aroma, taste and harmony found in the wine samples, and these parameters could receive maximum scores of 15, 30, 44 and 11, respectively [40].

### 3.9. Statistical Analysis

All experiments were repeated at least three times. All values are presented as the mean ± standard deviation (SD). Statistical analyses were performed using SPSS software (version 19.0; IBM Corporation, Armonk, NY, USA). The significance of the results was determined with an unpaired t-test or one-way ANOVA with Tukey’s test. For all graphs, bars with different symbols are significantly different at * *p* < 0.05, ** *p* < 0.01, and *** *p* < 0.001.

## 4. Conclusions

Compared with the Muscat Hamburg wines made by classical red and white-winemaking techniques, the results of this experiment demonstrated that the Muscat Hamburg wine made by carbonic maceration had significantly higher (*p* < 0.001) contents of esters (ethyl octanoate, ethyl hexanoate, ethyl decanoate, hexenyl acetate, isoamyl acetate and phenylethyl acetate) and terpenes ((±)-β-citronellol, linalool and limonene), which were the main aromatic compounds of the wine. Finally, the Muscat Hamburg wine prepared by carbonic maceration had a significantly better volatile profile and sensory evaluation scores relative to the wines prepared by the other two techniques. The results of this work will improve our understanding of the aromatic compounds, color and sensory properties of Muscat Hamburg wines. From this research, we can conclude that carbonic maceration is a better choice for making Muscat Hamburg wine and has great potential for broad future application. To date, research on wines prepared by carbonic maceration remains in the laboratory stage, and there are no wine produced by carbonic maceration in China. This is because Chinese wine consumers poorly understand the carbonic maceration technique, the quality of grapes are difficult to meet the large-scale production of carbonic maceration wines and the increased production cost also restricts the production of carbonic maceration wine. All carbonic macerated wines are imported from abroad. Therefore, we believe that the carbonic macerated Muscat Hamburg wine will meet the demands of wine consumers and the wine market in China.

## Figures and Tables

**Table 1:** General composition of Muscat Hamburg wines.

Composition of Wine	Type of Wine			Significance ^b^
	W	R	CM	
Alcoholicity (*v*/*v*, %)	12.05 ± 0.13	11.85 ± 0.16	11.75 ± 0.17	—
Reducing sugar (g/L)	2.67 ± 0.08	2.98 ± 0.11	3.11 ± 0.15	—
Free SO_2_ (mg/L)	5.42 ± 0.21	4.08 ± 0.25	5.33 ± 0.15	—
Total acidity ^a^ (g/L)	6.25 ± 0.15	6.45 ± 0.25	5.85 ± 0.16	—
pH value	3.41 ± 0.16	3.35 ± 0.24	3.65 ± 0.21	*
Dry extract (g/L)	25.35 ± 1.52	26.53 ± 2.16	26.15 ± 1.56	—

Results are the mean values for the three independent fermentations. ^a^ Measured as H_2_SO_4;_^b^ —; no significance; * *p* < 0.05.

**Table 2:** Colors of Muscat Hamburg wines from different winemaking technologies.

Color	Type of Wine		Significance *
	R	CM	
L*	96.41 ± 0.34	96.79 ± 0.42	—
C*	35.67 ± 0.28	41.26 ± 0.45	**
*h*	23.68 ± 0.12	24.14 ± 0.25	—
a*	37.65 ± 0.59	43.25 ± 0.45	**
b*	0.75 ± 0.12	0.84 ± 0.11	**

Results are the mean values for the three independent fermentations. * Significant data from red and carbonic macerated Muscat Hamburg wine; —; no significance; * *p* < 0.05; ** *p* < 0.01.

**Table 3:** The impact of different fermentation technologies on wine aroma compounds in Muscat Hamburg wine.

**Aroma Compound**	**Retention Index ^a^**	**Ion**	**Type of Wine (µg/L)**			**Odor Descriptors ^c^**	**Odorant Series**	**Threshold (µg/L)**	**Significance ^d^**
		***m*/*z*^b^**	**W**	**R**	**CM**				
**Alcohols**									
3-Methyl-1-butanol	1203	55	31.47 ± 1.23 *	42.74 ± 3.15 *	58.52 ± 1.47 *	Floral	2	30	***
2-Nonanol	1517	45	4.17 ± 0.27 *	4.56 ± 0.21 *	2.29 ± 0.08 *	Cucumber	4	58	***
Subtotal (µg/L)			35.64	47.3	60.81				
Subtotal (*w*/*w*, %)			0.84	1.31	0.95				
**Aldehydes and Ketones**									
(*E*)-2-Nonenal	1539	43	119.65 ± 9.23 *	70.34 ± 5.35 *	198.46 ± 16.32 *	Fatty	5	600	***
Octanal	1292	43	3.41 ± 0.13 *	—	4.41 ± 0.24 *	Fatty	5	15	—
Nonanal	1394	57	—	5.45 ± 0.25 *	4.47 ± 0.42 *	Green	4	1	—
Decanal	1499	43	11.86 ± 0.73 *	15.25 ± 1.07 *	12.54 ± 1.02 *	Grass	4	1000	***
β-Damascenone	1833	69	4.69 ± 0.26 *	6.39 ± 0.43 *	6.11 ± 0.41 *	Flowers, Apple, Rose, Honey	2, 3, 6	0.05	***
Subtotal (µg/L)			139.61	97.43	225.99				
Subtotal (*w*/*w*, %)			3.28	2.7	3.54				
**Terpenes**									
(−)-Rose oxide	1356	139	17.02 ± 0.71 *	6.86 ± 0.24 *	19.54 ± 0.83 *	Rose, Lychee	2, 6	0.2	***
(±)-β-Citronellol	1770	69	258.04 ± 21.59 *	40.18 ± 1.72 *	230.14 ± 11.8 *	Floral, Rose	2	18	***
4-Terpinenol	1575	81	1.78 ± 0.096 *	76.12 ± 7.19 *	2.25 ± 0.25 *	Sweet, Green, Citrus, Floral,	2, 3, 4, 6	250	***
Linalool	1547	71	179.21 ± 8.16 *	216.11 ± 8.31 *	269.45 ± 15.36 *	Flowery, Fruity	2, 6	15	***
Nerol	1805	69	11.6 ± 1.05 *	4.04 ± 0.16 *	18.04 ±1.62 *	Rose, Lime	2, 6	400	***
Limonene	1191	68	91.68 ± 6.52 *	26.68 ± 1.92 *	111.9 ± 5.19 *	Citrus-like, Fruity, Green	4, 6	10	***
Citral	1748	69	6.78 ± 0.52 *	—	2.68 ± 0.15 *	Floral, Lemon	2, 6	41	—
Caryophyllene	1581	93	—	9.18 ± 0.65 *	8.05 ± 0.58 *	Flowery	2	64000	—
Geraniol	1855	69	81.18 ± 5.32 *	13.76 ± 0.89 *	77.38 ± 6.32 *	Floral, Rose	2	30	***
α-Terpineol	1703	59	31.67 ± 2.14 *	12.17 ± 0.58 *	48.47 ± 2.86 *	Floral, Sweet	2, 3	1000	***
Subtotal (µg/L)			678.96	405.1	787.9				
Subtotal (*w*/*w*, %)			15.97	11.2	12.34				
**Acids**									
Hexanoic acid	1860	60	52.16 ± 2.62 *	42.35 ± 2.15 *	35.85 ± 1.21 *	Cheese, Fatty, Grass, Fruity	5, 6	140	***
*n*–Decanoic acid	2292	60	67.38 ± 3.15 *	53.24 ± 1.56 *	53.16 ± 1.36 *	Fatty	5	15,000	***
Subtotal (µg/L)			119.54	95.59	89.01				
Subtotal (*w*/*w*, %)			2.81	2.64	1.39				
**Esters**									
(*Z*)-3-Hexenyl acetate	1331	43	10.08 ± 0.69 *	3.83 ± 0.19 *	13.15 ± 0.73 *	Green, Apple, Grassy	4, 6	8	***
Ethyl butyrate	1047	43	21.25 ± 0.86 *	23.14 ± 1.34 *	24.23 ± 0.97 *	Fruity	6	20	***
Isoamyl acetate	1122	70	654.65 ± 5.35 *	581.24 ± 4.19 *	952.64 ± 8.17 *	Banana, Fruity, Sweet	3, 6	30	***
Ethyl caproate	1227	88	6.15 ± 0.27 *	5.01 ± 0.37 *	6.98 ± 0.19 *	Fruity, Banana	6	5	***
Ethyl hexanoate	1232	88	914.41 ± 5.32 *	785.63 ± 4.53 *	1077.56 ± 6.92 *	Green apple, Banana	6	14	***
Hexenyl acetate	1007	43	8.77 ± 0.75 *	10.16 ± 0.45 *	7.58 ± 0.34 *	Fruity	6	2	*
Ethyl heptanoate	1334	88	2.84 ± 0.06 *	3.91 ± 0.09 *	—	Pineapple, Green	4, 6	14	—
Methyl octanoate	1390	74	3.49 ± 0.21 *	4.16 ± 0.13 *	5.42 ± 0.17 *	Fruity, Green	4, 6	200	***
Ethyl octanoate	1437	88	724.43 ± 6.17 *	830.21 ± 7.21 *	1540.84 ± 11.17 *	Floral, Fruity, Banana, Pear	2, 6	5	***
Ethyl decanoate	1639	88	494.4 ± 4.13 *	293.25 ± 3.34 *	824.07 ± 6.24 *	Fruity	6	200	***
Ethyl 9-decenoate	1697	88	53.45 ± 2.36 *	31.26 ± 1.21 *	48.15 ± 2.17 *	Fruity, Fatty	5, 6	100	***
Phenylethyl acetate	1830	104	301.09 ± 2.07 *	341.08 ± 2.15 *	467.62 ± 1.67 *	Floral	2	250	***
Ethyl laurate	1848	88	82.89 ± 1.43 *	56.36 ± 0.32 *	253.02 ± 3.43 *	Fruity	6	1,500	***
Subtotal (µg/L)			3277.9	2969.24	5221.26				
Subtotal (*w*/*w*, %)			77.1	82.15	81.78				
Total (µg/L)			4251.65	3614.66	6384.97				

* Mean value and SD for three independent fermentation; — not data; ^a^ Kovats retention index was calculated based on an n-alkane series (C6–C24) on a poly(ethylene glycol) (PEG) column under the same chromatographic conditions. ^b^ The characteristic ion (*m*/*z*) was used for identifying the corresponding compound and evaluating their peak areas to avoid possible interference by other compounds. ^c^ 1, solvent; 2, floral; 3, sweet; 4, green; 5, fatty; 6, fruity. ^d^ * *p* < 0.05; *** *p* < 0.001.

**Table 4:** Sensory analysis of Muscat Hamburg wines produced by different winemaking techniques.

Attributes	Class	Type of Wine			Significance ^b^
		W	R	CM	
Visual analysis	Clarity (5)	4.5 ± 0.15	4.4 ± 0.15	4.6 ± 0.1	—
	Appearance (10)	8.5 ± 0.15	8.1 ± 0.2	8.2 ± 0.1	**
Aroma analysis	Aroma purity (6)	5.3 ± 0.1	5.1 ± 0.15	5.5 ± 0.15	***
	Aroma intensity (8)	6.8 ± 0.15	5.8 ± 0.15	7.3 ± 0.2	***
	Aroma quality (16)	12.1 ± 0.15	11.5 ± 0.15	13.5 ± 0.2	***
Taste analysis	Taste purity (6)	5.1 ± 0.1	4.6 ± 0.2	5.3 ± 0.2	***
	Taste intensity (8)	6.8 ± 0.1	6.7 ± 0.15	6.9 ± 0.2	—
	Taste prolongation (8)	6.6 ± 0.15	6.7 ± 0.1	7.1 ± 0.2	*
	Taste quality (22)	16.9 ± 0.1	16.1 ± 0.2	18.2 ± 0.2	***
Global evaluation	Harmony (11)	9.5 ± 0.15	8.6 ± 0.1	10.2 ± 0.15	***
Total ^a^	100	82.1 ± 0.3	77.6 ± 0.2	86.8 ± 0.4	***

^a^ > 86 = excellent; 81 – 85 = very good; 71 – 80 = good; 50 – 70 = average; < 50 = inadequate. ^b^ —; no significance; * *p* < 0.05; ** *p* < 0.01; *** *p* < 0.001.
